# Association Between Blood Pressure Control and Coronavirus Disease 2019 Outcomes in 45 418 Symptomatic Patients With Hypertension: An Observational Cohort Study

**DOI:** 10.1161/HYPERTENSIONAHA.120.16472

**Published:** 2020-12-16

**Authors:** James P. Sheppard, Brian D. Nicholson, Joseph Lee, Dylan McGagh, Julian Sherlock, Constantinos Koshiaris, Jason Oke, Nicholas R. Jones, William Hinton, Laura Armitage, Oliver Van Hecke, Sarah Lay-Flurrie, Clare R. Bankhead, Harshana Liyanage, John Williams, Filipa Ferreira, Michael D. Feher, Andrew J. Ashworth, Mark P. Joy, Simon de Lusignan, F.D. Richard Hobbs

**Affiliations:** 1 From the Nuffield Department of Primary Care Health Sciences, University of Oxford, United Kingdom (J.P.S., B.D.N., J.L., D.M., J.S., C.K., J.O., N.R.J., W.H., L.A., O.V.H., S.L.-F., C.R.B., H.L., J.W., F.F., M.D.F., M.P.J., S.d.L., F.D.R.H.); 2 Bonhard Medical, Bonhard House, Bo’ness, United Kingdom (A.J.A.).

**Keywords:** blood pressure, COVID-19, electronic health records, mortality, pandemic

## Abstract

Hypertension has been identified as a risk factor for coronavirus disease 2019 (COVID-19) and associated adverse outcomes. This study examined the association between preinfection blood pressure (BP) control and COVID-19 outcomes using data from 460 general practices in England. Eligible patients were adults with hypertension who were tested or diagnosed with COVID-19. BP control was defined by the most recent BP reading within 24 months of the index date (January 1, 2020). BP was defined as controlled (<130/80 mm Hg), raised (130/80–139/89 mm Hg), stage 1 uncontrolled (140/90–159/99 mm Hg), or stage 2 uncontrolled (≥160/100 mm Hg). The primary outcome was death within 28 days of COVID-19 diagnosis. Secondary outcomes were COVID-19 diagnosis and COVID-19–related hospital admission. Multivariable logistic regression was used to examine the association between BP control and outcomes. Of the 45 418 patients (mean age, 67 years; 44.7% male) included, 11 950 (26.3%) had controlled BP. These patients were older, had more comorbidities, and had been diagnosed with hypertension for longer. A total of 4277 patients (9.4%) were diagnosed with COVID-19 and 877 died within 28 days. Individuals with stage 1 uncontrolled BP had lower odds of COVID-19 death (odds ratio, 0.76 [95% CI, 0.62–0.92]) compared with patients with well-controlled BP. There was no association between BP control and COVID-19 diagnosis or hospitalization. These findings suggest BP control may be associated with worse COVID-19 outcomes, possibly due to these patients having more advanced atherosclerosis and target organ damage. Such patients may need to consider adhering to stricter social distancing, to limit the impact of COVID-19 as future waves of the pandemic occur.

## Abstract 2

Supplemental Digital Content is available in the text.

Coronavirus disease 2019 (COVID-19) is caused by severe acute respiratory syndrome coronavirus 2 (SARS-CoV-2) and has spread rapidly across the globe resulting in significant restrictions on daily life for millions, serious health complications, and death.^1^ Over the past 6 months, studies have identified common comorbidities in patients with COVID-19, including hypertension and cardiovascular disease,^2^ which increase the likelihood of serious complications such as hospitalization and death.^3–5^

One initial proposed explanation for the association between hypertension and COVID-19 was that the SARS-CoV-2 virus enters cells in the lung via ACE2 (angiotensin-converting enzyme 2) receptors.^6^ People with hypertension are more likely to be prescribed medications (such as angiotensin-converting enzyme inhibitors or angiotensin II receptor blockers) that upregulate expression of ACE2 and, therefore, increase patient susceptibility to SARS-CoV-2 cell entry.^7,8^ However, this theory has since been dismissed^9,10^ with some more recent studies suggesting that the prescription of renin-angiotensin system medications may in fact protect against SARS-CoV-2 infection^11^ and COVID-19–related death.^12^

Most recently, a study based on data from hospitals in China suggested that it is higher blood pressure, not specific medication use, which is an important independent risk factor for complications, such as heart failure, in patients with COVID-19 with hypertension.^13^ Establishing whether this is also the case for hypertensive patients living in the community is important because the focus on routine chronic disease management has reduced during the pandemic.^14^ Based on previous studies,^3–5,13,15–17^ we hypothesized that uncontrolled blood pressure would be associated with worse COVID-19 outcomes for hypertensive patients with suspected COVID-19. We used the electronic health records from primary care to test this hypothesis and examined the association between blood pressure control and SARS-CoV-2 infection, COVID-19–related hospitalization, and death.

## Methods

### Design

This study used a retrospective observational cohort study design, utilizing electronic health records from general practices in England contributing to the Oxford Royal College of General Practitioners Clinical Informatics Digital Hub (ORCHID).^18–20^ The ORCHID hub is representative of patients attending English primary care across urban and nonurban practices.^19^ The protocol for this study was approved by Royal College of General Practitioners Research Surveillance Centre (RCGP RSC) scientific advisory committee and received ethical approval from the University of Oxford, Medical Sciences Interdivisional Research Ethics Committee (ref: R54893/RE001). Because of the sensitive nature of the data collected for this study, requests to access the dataset from qualified researchers trained in human subject confidentiality protocols may be sent to the RCGP RSC at MedicalDirectorRSC@rcgp.org.uk.

### Study Population

This study examined patients aged 18 years and older, with a coded history of hypertension and registered at general practices in England contributing to ORCHID. Early on in the pandemic, many people are thought to have contracted COVID-19 without realizing or being tested.^21^ The present analyses, therefore, focused on individuals tested for or who had a clinical diagnosis of COVID-19 to minimize bias from incomplete outcome ascertainment (ie, to avoid missing patients who experienced relevant outcomes but were not tested for COVID-19). This information was derived from a newly developed COVID-19 ontology^22^ which uses coded information in an individual’s electronic health record to determine their COVID-19 status. Patients were classified as either not diagnosed with COVID-19 (negative virology test for SARS-CoV-2) or diagnosed with COVID-19 (based on a diagnostic code for COVID-19 or a positive virology test for SARS-CoV-2). All patients entered the cohort on the January 1, 2020 (index date) and were followed until August 31, 2020.

### Exposures

The primary exposure of interest in this study was blood pressure control at the index date (January 1, 2020). This was defined according to the most recently recorded blood pressure in a patient’s electronic health record (within 24 months of the index date). Because readings were taken from routine electronic health records, the exact method of measurement would have varied between patients and was not captured in the record itself. A period of up to 24 months was chosen to maximize the number of participants that could be included in the complete case analysis. Blood pressure control was specified as a categorical variable according to clinical guidelines,^23^ consisting of controlled (readings <130/80 mm Hg), raised (readings between 130/80 and 139/89 mm Hg), stage 1 uncontrolled (readings between 140/90 and 159/99 mm Hg), and stage 2 uncontrolled blood pressure (readings ≥160/100 mm Hg). Sensitivity analyses examined blood pressure control defined as a binary variable (readings ±140/90 mm Hg), using systolic blood pressure as a continuous variable and using a categorical variable defined by estimating the mean of up to 25 readings taken within the 24 months before the index date.

### Outcomes

The primary outcome in this analysis was death within 28 days of a COVID-19 diagnosis recorded in the patient’s electronic health record, as per the current definition of COVID-19–related death in the United Kingdom.^24^ This conservative definition was used because the longer the interval between diagnosis and death, the more likely non-COVID deaths could occur and be misclassified as being COVID-related. Secondary outcomes were COVID-19 diagnosis and hospital admission related to COVID-19. The latter was defined as either a hospital admission within 28 days of COVID-19 diagnosis or a COVID-19 diagnostic code being entered into the medical records after hospital admission but before discharge. No linked secondary care or death registry data were available for this analysis, so all outcomes were based on codes entered into the primary care electronic health record.

### Covariates

All analyses were adjusted for covariates thought to predict COVID-19 outcomes as determined by the previous literature.^1,5,11^ These were age at index date, sex, ethnicity, indices of social deprivation (indices of multiple deprivation quintile), number of people within the household, smoking status (current, ex, or never smoked), coded as being on the COVID-19 shielding list (due to comorbidities), and most recent measure of body mass index, specified as a continuous variable. Those with missing ethnicity or smoking status were classed as unknown ethnicity or nonsmokers, respectively. Comorbidities were defined as those present before the index date including asthma, cancer, chronic lung disease, chronic obstructive pulmonary disease, chronic kidney disease, diabetes, previous myocardial infarction, stroke, or transient ischemic attack. Models were adjusted for the presence of a prescribed cardiovascular medication at the index date. This included all blood pressure-lowering medications and statins entered as individual drug classes. Each model was also adjusted for the date at which COVID-19 was first suspected using codes from the COVID-19 ontology.^22^

### Statistical Analysis

Descriptive statistics were used to define the characteristics of the study population. Multivariable logistic regression was used to examine the association between blood pressure control and COVID-19 outcomes. This model was chosen since follow-up was short (8 months), and so the likelihood of censoring due to competing risks or loss to follow-up was low. All models were adjusted for the covariates described above, but interaction terms between covariates were not included. Missing data for indices of multiple deprivation, body mass index, blood pressure, and smoking status were low (<5%), so no attempts were made to impute missing values and a complete case analysis was conducted.

Subgroup analyses were undertaken to examine the association between blood pressure control and COVID-19–related death in young versus older adults (18–69 years versus 70+ years), those with diabetes, chronic kidney disease, cardiovascular disease, and those prescribed renin-angiotensin system medications versus those prescribed other blood pressure-lowering medications. Because the availability of testing changed significantly during the study period (and, therefore, the types of patients receiving such tests might also have changed), further analyses were conducted according to the time period in which patients were first suspected of COVID-19 (ie, January to March, April to June, and July to Aug 2020). Post hoc analyses examined the primary outcome in those prescribed 0 to 2 antihypertensives versus those prescribed 3+ antihypertensive medications.

All data are presented as means, medians, or odds ratios (ORs) with 95% CI or interquartile range. Analyses were conducted using STATA 14.2 (StataCorp, TX).

## Results

The ORCHID database included a total of 4 101 459 active patients, from 460 general practices. A total of 45 418 patients had a history of hypertension, had a blood pressure reading in the preceding 24 months (40 645 [89.5%] had a reading within 12 months of the index date), and were tested for or diagnosed with COVID-19. Overall, patients were aged 67.3±16.0 years, 44.7% were male, and 75.6% were of white ethnicity (Table 1). The median household size was 2 people (interquartile range, 1–4), and 15.9% of patients lived in regions with the highest levels of deprivation (fifth quintile of indices of multiple deprivation).

**Table 1.**
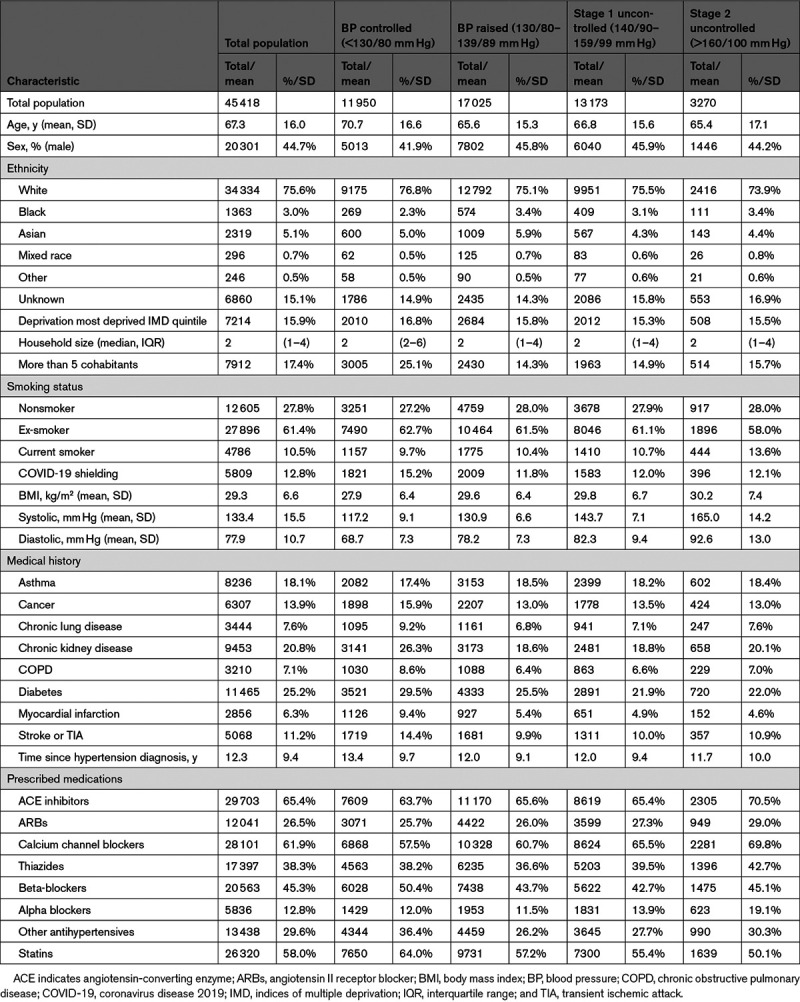
Characteristics of Patients With a History of Hypertension at the Index Date

There were 11 950 (26.3%) patients with controlled blood pressure, 17 025 (37.5%) with moderately raised blood pressure, and 16 443 (36.2%) with uncontrolled blood pressure (stage ≥1). A higher proportion of patients with raised and uncontrolled blood pressure were of black ethnicity but fewer were coded with COVID-19 shielding status (Table 1). Patients with controlled blood pressure were older (71 years versus 65–67 years) and had been diagnosed with hypertension for at least 1.4 years longer than those with raised or uncontrolled blood pressure. They also had more comorbidities, including chronic kidney disease (26.3%), chronic obstructive pulmonary disease (8.6%), diabetes (29.5%), history of myocardial infarction (9.4%), and stroke or transient ischemic attack (14.4%).

A total of 4277 (9.4%) were diagnosed with COVID-19 (including 3025 [6.7%] with a positive virology test for SARS-CoV-2; Table 2). Across the study population, there were 273 (0.6%) COVID-19–related hospitalizations and 877 (1.9%) COVID-19–related deaths.

**Table 2.**
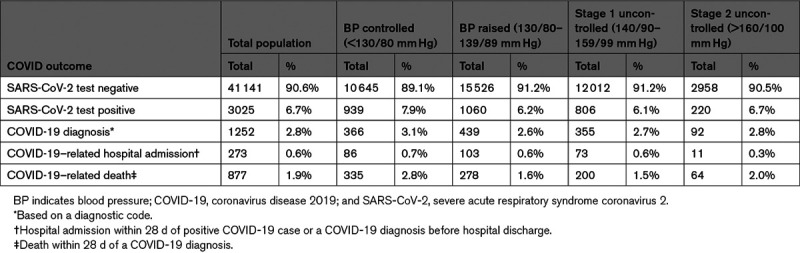
Patients Being Investigated for COVID and Experiencing Outcomes During Follow-Up

### Primary Outcome

**Figure 1.**
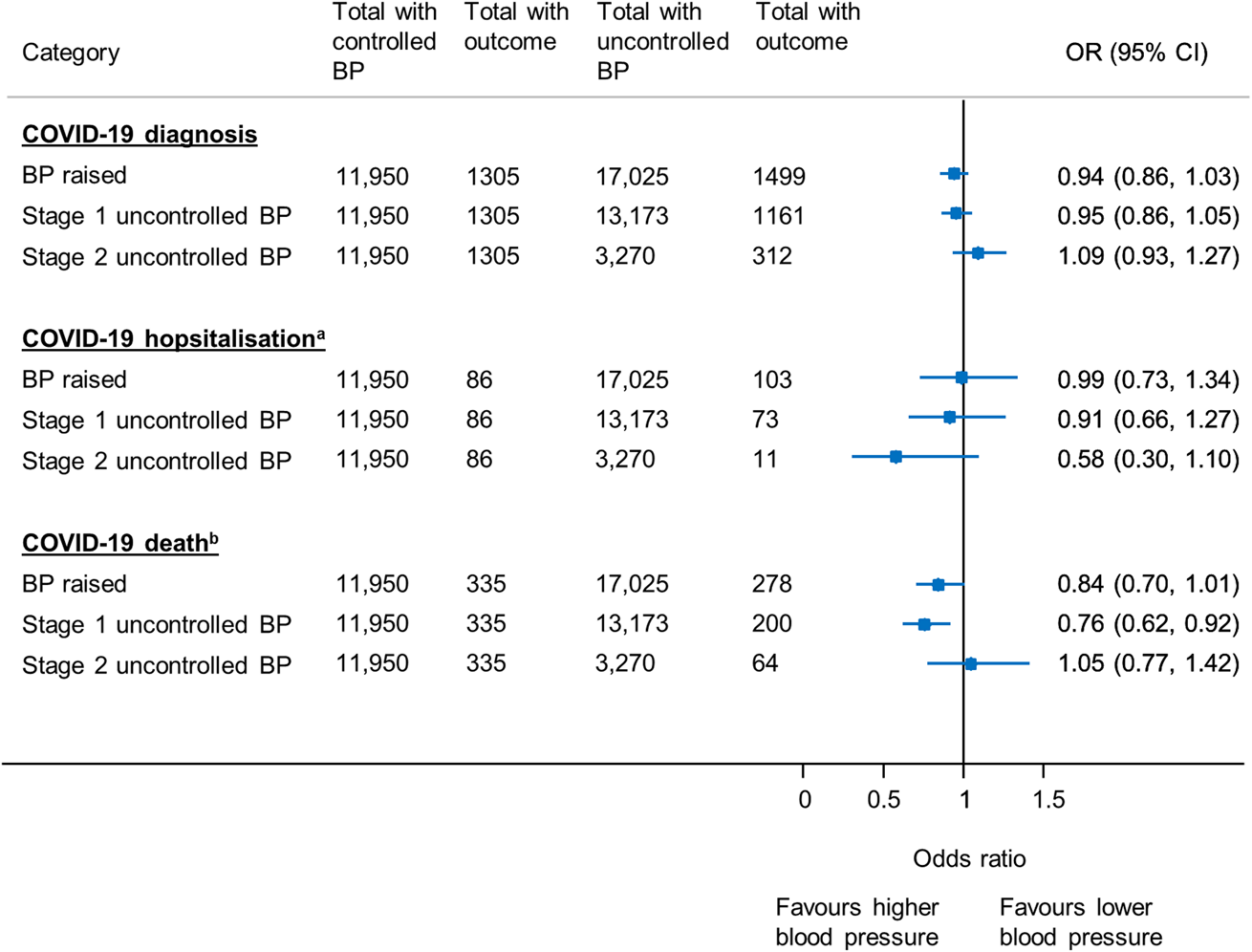
**Primary analysis showing the association between blood pressure (BP) control and coronavirus disease 2019 (COVID-19) diagnosis, COVID-19–related hospitalization and death.** Models adjusted for age, sex, ethnicity, deprivation, household size, body mass index, smoking status, COVID-19 shielding status, date of suspected COVID-19 diagnosis, diabetes, chronic kidney disease, previous stroke, previous transient ischemic attack, previous myocardial infarction, chronic lung disease, asthma, chronic obstructive pulmonary disease, cancer, antihypertensive, and statin prescription. OR indicates odds ratio. ^a^Hospital admission within 28 d of positive COVID-19 case or a COVID-19 diagnosis before hospital discharge. ^b^Death within 28 d of a COVID-19 diagnosis.

In multivariable analyses adjusting for all covariates, individuals with stage 1 uncontrolled blood pressure had lower odds of COVID-19–related death (OR, 0.76 [95% CI, 0.62–0.92]) compared with patients with well-controlled blood pressure (<130/80 mm Hg; Figure 1). Moderately raised blood pressure and stage 2 or above uncontrolled blood pressure were not associated with COVID-19–related death (raised BP, OR, 0.84 [95% CI, 0.70–1.01]; stage ≥2 uncontrolled BP, OR, 1.05 [95% CI, 0.77–1.42]; Figure 1). Increasing age, male sex, Asian, or other ethnicity (compared with White), increasing deprivation, living in a multiperson household, being an ex-smoker, and having diabetes were all significant predictors of COVID-19–related death (Table S1 in the Data Supplement).

### Secondary Outcomes

There was no association between moderately raised or uncontrolled blood pressure and COVID-19 diagnosis (raised BP, OR, 0.94 [95% CI, 0.86–1.03]; stage 1 uncontrolled BP, OR, 0.95 [95% CI, 0.86–1.05]; stage 2 uncontrolled BP, OR, 1.09 [95% CI, 0.93–1.27]); Figure 1). Further analyses focusing on COVID-19–related hospital admission found no association with blood pressure control (raised BP, OR, 0.99 [95% CI, 0.73–1.34]; stage 1 uncontrolled BP, OR, 0.91 [95% CI, 0.66–1.27]; stage ≥2 uncontrolled BP, OR, 0.58 [95% CI, 0.30–1.10]; Figure 1).

### Sensitivity and Subgroup Analyses

**Figure 2.**
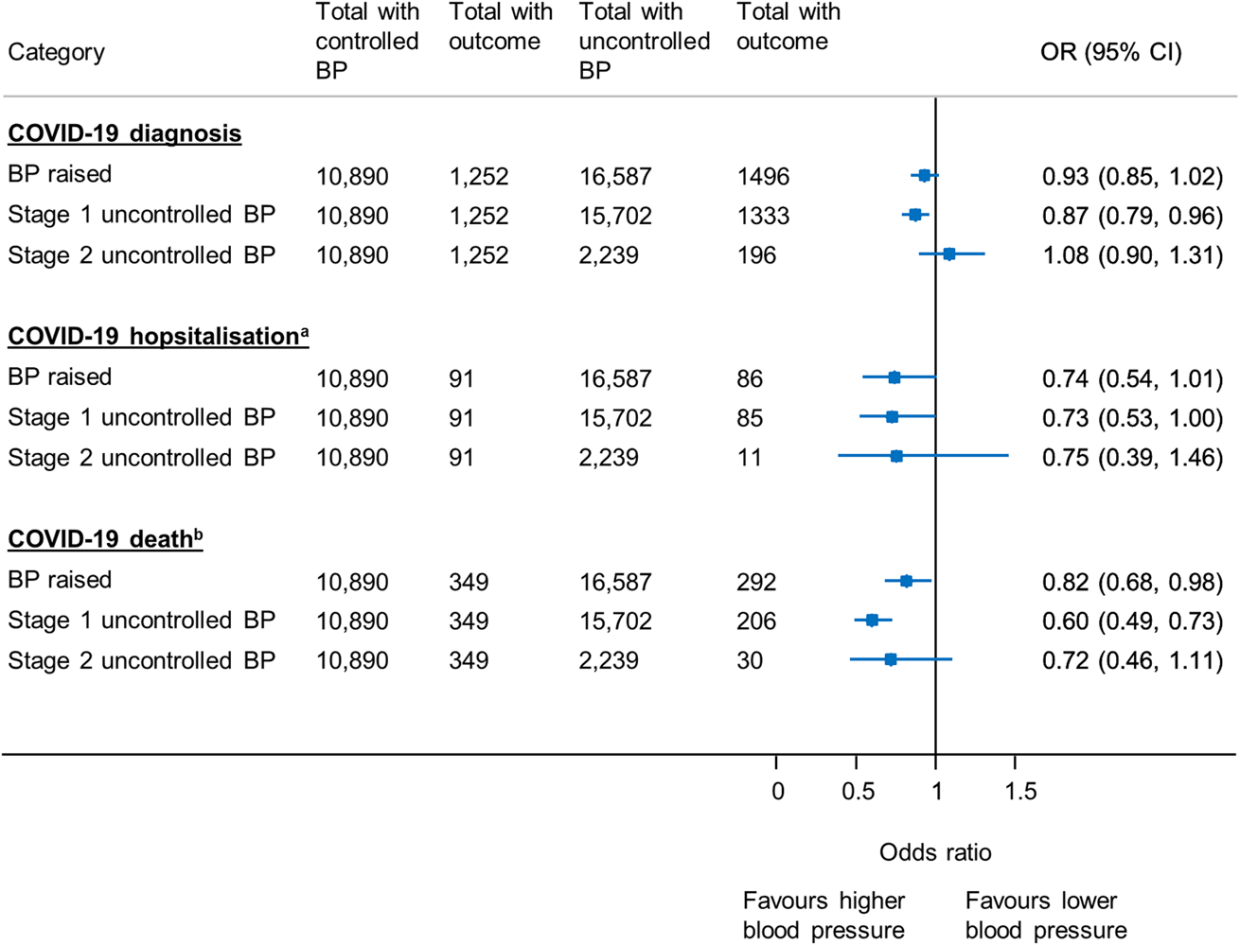
**Sensitivity analyses examining the association between blood pressure (BP) control (defined by the mean of all BP readings in the preceding 2 y) and coronavirus disease 2019 (COVID-19) diagnosis, COVID-19–related hospitalization, and death.** Models adjusted for age, sex, ethnicity, deprivation, household size, body mass index, smoking status, COVID-19 shielding status, date of suspected COVID-19 diagnosis, diabetes, chronic kidney disease, previous stroke, previous transient ischemic attack, previous myocardial infarction, chronic lung disease, asthma, chronic obstructive pulmonary disease, cancer, antihypertensive, and statin prescription. OR indicates odds ratio. ^a^Hospital admission within 28 d of positive COVID-19 case or a COVID-19 diagnosis before hospital discharge. ^b^Death within 28 d of a COVID-19 diagnosis.

Patients had a median of 4 blood pressure readings (interquartile range, 3–7) in the 24 months preceding the index date. In analyses based on the average of these readings, moderately raised blood pressure and stage 1 uncontrolled blood pressure were associated with lower odds of COVID-19–related death, compared with patients with well-controlled blood pressure (Figure 2). Stage 1 uncontrolled blood pressure was also associated with lower odds of COVID-19 diagnosis.

**Figure 3.**
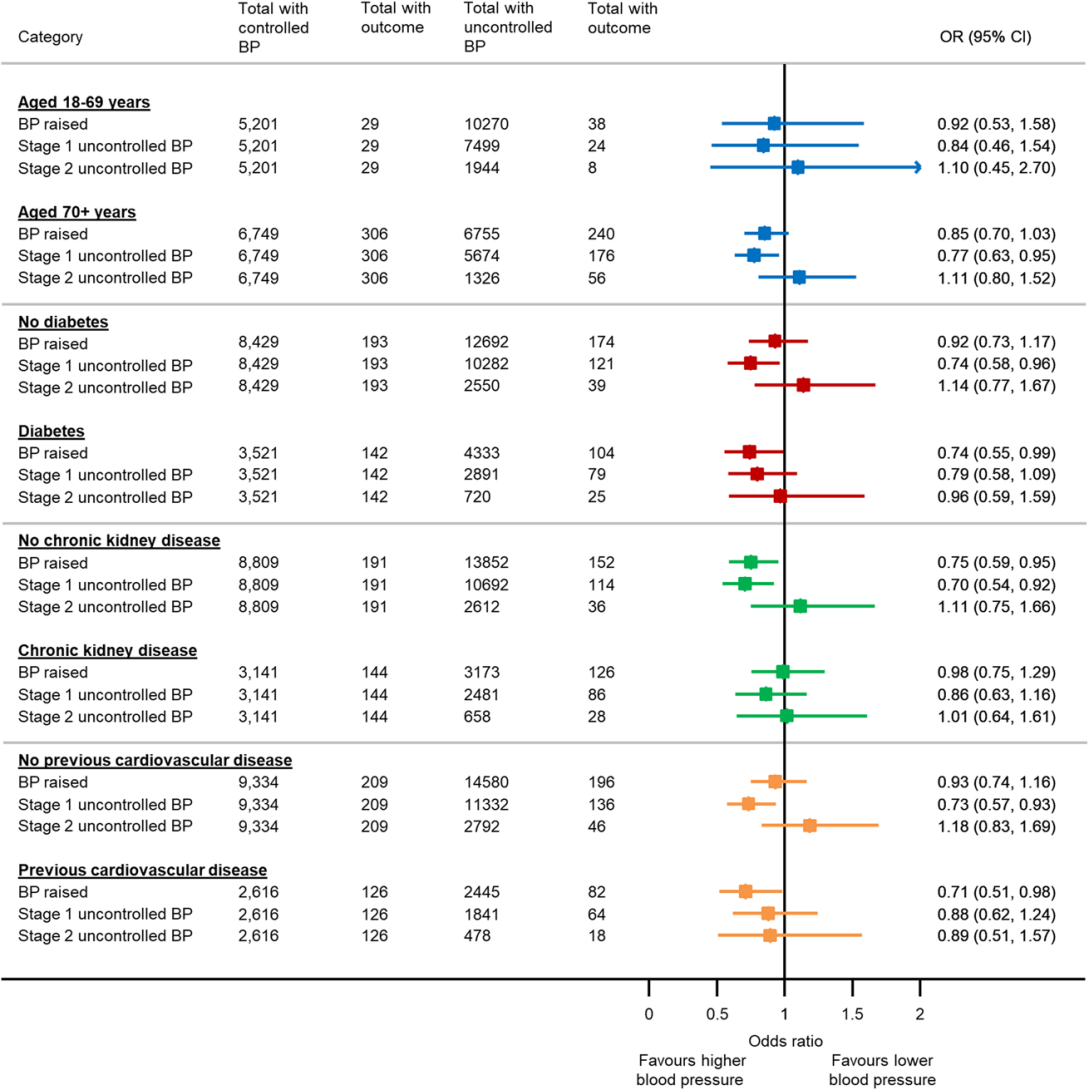
**Subgroup analyses examining the association between blood pressure (BP) control and coronavirus disease 2019 (COVID-19)–related death according to age and selected comorbidities.** Models adjusted for age, sex, ethnicity, deprivation, household size, body mass index, smoking status, COVID-19 shielding status, date of suspected COVID-19 diagnosis, diabetes, chronic kidney disease, previous stroke, previous transient ischemic attack, previous myocardial infarction, chronic lung disease. OR indicates odds ratio.

Sensitivity analyses including blood pressure control as a binary outcome and systolic blood pressure as a continuous variable confirmed the findings of the primary analysis showing a limited association between uncontrolled blood pressure and COVID-19–related death (Table S2, Appendix). The association between stage 1 uncontrolled blood pressure and COVID-19–related death was only present in older patients (70+ years), those without a history of diabetes, chronic kidney disease, or cardiovascular disease (Figure 3), and those prescribed renin-angiotensin system medications (Table S3). The findings of the primary analysis were not altered by the time period of first suspected SARS-CoV-2 infection (Table S3). Post hoc analyses showed the association between stage 1 uncontrolled blood pressure and less COVID-19–related death was only present in patients prescribed three or more antihypertensive medications (Table S4).

## Discussion

### Summary of Main Findings

This is the largest study of COVID-19 outcomes in community-dwelling patients with hypertension conducted to date. Across 45 418 patients with hypertension and suspected COVID-19, those with recent stage 1 uncontrolled blood pressure had lower odds of COVID-19–related death compared with patients with well-controlled blood pressure. There was no association between moderately raised blood pressure or stage 2 uncontrolled blood pressure and COVID-19–related death. These findings were robust to sensitivity analyses and contrary to our hypothesis that raised or uncontrolled blood pressure would be associated with worse COVID-19 outcomes. In analyses defining blood pressure control over a longer period of time (across 2 years before the index date), both moderately raised and stage 1 uncontrolled blood pressure were associated with lower odds of COVID-19–related death.

Patients with strictly controlled blood pressure were older, had more comorbidities, and had been diagnosed with hypertension for longer. A possible explanation for the observed associations is that patients with strict blood pressure control had more advanced atherosclerosis compared with those with moderately raised and uncontrolled blood pressure. This interpretation is supported by our observation of a higher prevalence of target organ damage (including chronic kidney disease, myocardial infarction, stroke, and transient ischemic attack) in those with strictly controlled hypertension and other data, suggesting that COVID-19 and cardiovascular disease have a bidirectional relationship.^2^ These findings suggest those with long-term controlled blood pressure may need to consider stricter social distancing to limit the impact of COVID-19 as future waves of the pandemic occur.

### Strengths and Limitations

This is the largest study examining the association between blood pressure and COVID-19 outcomes conducted in community-dwelling patients with hypertension. The ORCHID hub^20^ is capable of weekly data downloads permitting some of the most timely and up to date analysis of primary care data in the world. Data from secondary care are not available in such a timely manner in the United Kingdom, and for this analysis, it was not possible to link primary care data to hospital databases or the national death registry. As a result, it is possible that the total number of COVID-19–related outcomes (particularly hospital admissions, which were lower than anticipated) may have been underestimated. The quality of coding of hospitalization in primary care records is likely to vary between primary care providers,^25^ and so we implemented a strict definition of COVID-19–related admissions (within 28 days of diagnosis) which may also have resulted in some relevant outcomes being missed.

Furthermore, because the data used here included patients tested for COVID-19 within a week of conducting the analysis, some recently infected patients may have gone on to have hospital admissions or die but these outcomes would not have been captured in this analysis. We would not expect systematic differences in the recording of COVID-19 outcomes depending on an individual’s blood pressure level, so the impact of these potential missing outcome data is likely to be small.

This was an observational study using data from routine electronic health records. As such, the main exposure (blood pressure) was based on measurements taken in routine clinical practice. One-off measurements taken in this setting may not accurately reflect the underlying blood pressure of each patient, leading to the potential for classification bias. However, sensitivity analyses examining blood pressure based on the mean of up to 25 readings taken across the preceding 24-month period showed similar findings, suggesting this bias may not have had an important influence on the results.

People with very low blood pressure, on multiple antihypertensive medications, have been shown to be at increased risk of mortality.^26^ However, although some of these may have been captured in the strictly controlled blood pressure group, we do not think there were sufficient numbers to notably alter the findings.

Finally, rates of COVID-19 testing have changed significantly during the pandemic which may have affected ascertainment of COVID-19 cases and the type of patient included in the analysis cohort (potential selection bias). To mitigate this, we implemented a COVID-19 ontology^22^ which allowed us to identify and include patients with coded COVID-19 diagnosis but who did not receive a confirmatory virology test. Date of first suspected SARS-CoV-2 infection was adjusted in the analysis and subgroup analyses by time period and did not show a difference in the association between blood pressure control and COVID-19 outcomes, suggesting any changes in the ascertainment of cases or potential selection bias did not have a large impact on the main findings.

### Comparison With Previous Literature

Hypertension has previously been shown to be a risk factor for worse COVID-19 outcomes,^6^ and there are some data to suggest that patients admitted to intensive care with COVID-19 have higher blood pressure compared with those who do not have COVID-19.^15,16^ A recent study from China showed that patients with higher blood pressure during admission to hospital have a higher risk of heart failure but not mortality or intensive care unit admission.^13^ In contrast, the present study found an inverse relationship between recent blood pressure control and COVID-19–related death. This relationship was robust to sensitivity analyses examining blood pressure as a continuous variable and defining blood pressure control using the average of readings taken across the preceding 2 years. The fact that our study focused on blood pressure control before COVID-19 diagnosis, rather than during hospitalization for an infection may explain these discrepant results.

Given the limited data on COVID-19 and related risk factors,^3–5,17^ this finding is entirely novel and not easily explained. It may be a chance statistical finding due to multiple hypothesis testing and future studies should look to confirm the relationship observed in these data. It is also possible that other important risk factors were present in the group with controlled blood pressure (such as congestive heart failure), but not adequately adjusted for in the analysis. Indeed, when this study was conceived, very little was known about what conditions and medications modify the risk of COVID-19–related death and so it was not possible to include them in the dataset and adjust for them in the analysis.

Another possible explanation is that blood pressure control, as defined in the present analyses, is a surrogate marker for underlying atherosclerosis, which in turn is associated with increased odds of COVID-19 outcomes.^2^ This would seem to be backed up by the observation that individuals with controlled blood pressure were older, had more comorbidities (including target organ damage), and had been diagnosed with hypertension for longer. In the United Kingdom, blood pressure treatment targets are lower for people with comorbidities, such as diabetes and chronic kidney disease,^27^ and physicians may be more likely to treat hypertension aggressively in high-risk patients with established cardiovascular disease. This would lead to individuals at higher risk of COVID-19–related outcomes being more likely to have controlled blood pressure.

### Implications for Clinical Practice

Establishing the association between hypertension control and COVID-19 outcomes has important implications for ongoing management, particularly as future waves of the pandemic occur. This analysis suggests that recent, poorly controlled blood pressure does not carry an increased risk of COVID-19–related complications, beyond that of the underlying hypertension. This may be reassuring given that chronic disease management has been deprioritized during the pandemic.^14^ However, high blood pressure remains a strong risk factor for cardiovascular disease, including stroke,^28^ the consequences of which can be comparable or worse than those of COVID-19. Thus, although stricter blood pressure control in patients with hypertension does not appear to reduce the risk of COVID-19 complications, physicians should continue to ensure adequate blood pressure control to prevent long-term outcomes, such as stroke. They may also attempt to identify and monitor individuals with advanced atherosclerosis, perhaps focusing on those who have had well-controlled blood pressure for a longer period of time or who have been diagnosed with hypertension for many years. These patients may need to consider adhering to stricter social distancing, to limit the impact of COVID-19 as future waves of the pandemic occur.

### Perspectives

This study found little evidence to support the hypothesis that stricter blood pressure control reduces the risk of complications from COVID-19 in patients with hypertension. Future studies should look to confirm the observation that blood pressure control is associated with increased odds of COVID-19–related death. This may be due to underlying atherosclerosis in these patients and physicians should monitor such patients carefully, as they may need to adhere to stricter social distancing to limit the impact of COVID-19 in future waves of the pandemic.

## Sources of Funding

This piece of work was not specifically funded but used data from the Oxford Royal College of General Practitioners Clinical Informatics Digital Hub (ORCHID) hub, which is partially supported by the University of Oxford Medical Sciences Division Urgent coronavirus disease (COVID) Fund and a discretionary award from the Primary Care Research Trust. All COVID-19 research conducted within ORCHID is supported by Public Health England, the National Institute for Health Research (NIHR) Oxford and Thames Valley Applied Research Collaboration. J.P. Sheppard is supported by the Wellcome Trust/Royal Society via a Sir Henry Dale Fellowship (ref: 211182/Z/18/Z) and the NIHR Oxford Biomedical Research Centre. N.R. Jones is supported by a Wellcome Trust Doctoral Research Fellowship (ref: 203921/Z/16/Z). F.D. Richard Hobbs acknowledges part-funding from the NIHR School for Primary Care Research, the NIHR Collaboration for Leadership in Health Research and Care (CLARHC) Oxford, the NIHR Oxford Biomedical Research Centre (Biomedical Research Centre, Oxford University Hospitals NHS Foundation Trust), and the NIHR Oxford Medtech and In-Vitro Diagnostics Co-operative (Oxford Meditech and In-Vitro Diagnostics Co-operative). C.R. Bankhead is supported by the NIHR Oxford Biomedical Research Centre and the NIHR Thames Valley Applied Research Collaborative.

## Disclosures

None.

## Supplementary Material



## Novelty and Significance

### What Is New?

- This is the largest study of coronavirus disease 2019 (COVID-19)–related outcomes in community-dwelling patients with hypertension.
- Findings show that patients with recent stage 1 uncontrolled blood pressure had lower odds of COVID-19 death compared with patients with well-controlled blood pressure.

### What Is Relevant?

- Patients with controlled blood pressure were older, had more comorbidities, and had been diagnosed with hypertension for longer.
- These patients may have more advanced underlying atherosclerosis and target organ damage leading to greater risk of COVID-19 complications.

### Summary

Hypertensive patients with long-term blood pressure control may need to consider adhering to stricter social distancing to limit the impact of COVID-19 in future waves of the pandemic.
