# Supplementary material for: Association Between Blood Pressure Control and Coronavirus Disease 2019 Outcomes in 45 418 Symptomatic Patients With Hypertension: An Observational Cohort Study
Source: Hypertension. 2020 Dec 16;77(3):846–55. doi: 10.1161/HYPERTENSIONAHA.120.16472 (PMC7884248; doi:10.1161/HYPERTENSIONAHA.120.16472)
Supplement: Supplementary file 1 [file hyp-77-846-s001.doc]

**The association between blood pressure control and Coronavirus Disease 2019 outcomes in 45,418 symptomatic patients with hypertension: An observational cohort study**

**Online Supplement**

James P. Sheppard, Brian D. Nicholson, Joseph Lee, Dylan McGagh, Julian Sherlock, Constantinos Koshiaris, Jason Oke, Nicholas R. Jones, William Hinton, Laura Armitage, Oliver Van Hecke, Sarah Lay-Flurrie, Clare R. Bankhead, Harshana Liyanage, John Williams, Filipa Ferreira, Michael D. Feher, Andrew J. Ashworth, Mark P. Joy, Simon de Lusignan, FD Richard Hobbs

**Contents**

1. **Table S1.** Full model for the primary analysis showing the association between blood pressure control and COVID-19 death*, adjusting for other covariates
2. **Table S2.** Sensitivity analyses examining outcomes with the exposure defined as a binary and continuous variable
3. **Table S2.** Subgroup analyses examining the association between blood pressure control and COVID-19 death* according to RAS exposure and time of infection
4. **Table S4.** Post-hoc subgroup analyses examining the association between blood pressure control and COVID-19 death* according to number of antihypertensive medications prescribed

**Table S1.** Full model for the primary analysis showing the association between blood pressure control and COVID-19 death*, adjusting for other covariates

| **Covariate** | **Odds Ratio** | **95% confidence interval** | |
| --- | --- | --- | --- |
|  |  | **Lower** | **Upper** |
| **Blood pressure (BP) category** (ref = controlled BP) |  |  |  |
| BP moderately raised | 0.84 | 0.70 | 1.01 |
| Grade 1 uncontrolled BP | 0.76 | 0.62 | 0.92 |
| Grade 2 uncontrolled BP | 1.05 | 0.77 | 1.42 |
| **Age** (continuous) | 1.07 | 1.06 | 1.08 |
| **Sex** (ref = female) | 1.63 | 1.38 | 1.92 |
| **Ethnicity** (ref = white) |  |  |  |
| Asian | 1.81 | 1.24 | 2.65 |
| Black | 1.36 | 0.86 | 2.13 |
| Other ethnicity | 2.52 | 1.14 | 5.55 |
| Unknown ethnicity | 0.89 | 0.72 | 1.10 |
| **Indices of multiple deprivation** (ref = 5^th^ quintile [most deprived]) | | |  |
| 1^st^ quintile (least deprived) | 0.42 | 0.33 | 0.54 |
| 2^nd^ quintile | 0.64 | 0.50 | 0.81 |
| 3^rd^ quintile | 0.72 | 0.57 | 0.91 |
| 4^th^ quintile | 0.67 | 0.52 | 0.86 |
| **Household size** (continuous) | 1.01 | 1.01 | 1.02 |
| **Body mass index** (continuous) | 1.00 | 0.98 | 1.01 |
| **Smoking status** (ref = non-smoker) |  |  |  |
| Current smoker | 1.07 | 0.75 | 1.53 |
| Ex-smoker | 1.22 | 1.01 | 1.47 |
| **COVID-19 shielding status** (ref = no) | 1.05 | 0.87 | 1.28 |
| **Date of COVID-19 diagnosis** (continuous) | 0.96 | 0.96 | 0.97 |
| **Co-morbidities** |  |  |  |
| Diabetes | 1.37 | 1.16 | 1.61 |
| Chronic kidney disease | 1.07 | 0.91 | 1.26 |
| Stroke or transient ischaemic attack | 1.16 | 0.96 | 1.40 |
| Myocardial infarction | 1.23 | 0.98 | 1.54 |
| Chronic lung disease | 1.07 | 0.55 | 2.08 |
| Asthma | 0.75 | 0.60 | 0.94 |
| Chronic Obstructive Pulmonary Disease | 1.02 | 0.51 | 2.05 |
| Cancer | 1.10 | 0.92 | 1.32 |
| **Prescribed medications** |  |  |  |
| Angiotensin converting enzyme (ACE) inhibitor | 1.06 | 0.89 | 1.27 |
| Angiotensin II receptor blocker (ARB) | 0.99 | 0.84 | 1.17 |
| Alpha-blocker | 1.01 | 0.83 | 1.22 |
| Beta-blocker | 1.04 | 0.88 | 1.23 |
| Calcium channel blockers | 1.09 | 0.91 | 1.30 |
| Thiazide or thiazide-like diuretic | 0.96 | 0.82 | 1.13 |
| Loop diuretic | 1.41 | 1.19 | 1.67 |
| Potassium sparing diuretic | 1.07 | 0.84 | 1.36 |
| Centrally acting antihypertensive | 1.16 | 0.77 | 1.73 |
| Vasodilator antiHypertensive | 0.88 | 0.62 | 1.27 |
| Combination therapy (ACE + other) | 1.46 | 0.28 | 7.75 |
| Combination therapy (ARB + other) | 1.54 | 0.55 | 4.28 |
| Statin | 0.93 | 0.77 | 1.12 |

*Death within 28 days of a COVID-19 diagnosis.

**Table S2.** Sensitivity analyses examining outcomes with the exposure defined as a binary and continuous variable

| **Exposure and outcome** | **Total with controlled BP** | **Total with outcome** | **Total with uncontrolled BP** | **Total with outcome** | **Odds ratio** | **95% CI lower** | **95% CI upper** |
| --- | --- | --- | --- | --- | --- | --- | --- |
| *BP uncontrolled (+/- 140/90 mmHg)* | | | | | | | |
| COVID-19 diagnosis | 28,975 | 2,804 | 16,443 | 1,473 | 1.01 | 0.94 | 1.10 |
| COVID-19 hospitalisation^a^ | 28,975 | 189 | 16,443 | 84 | 0.86 | 0.66 | 1.12 |
| COVID-19 death^b^ | 28,975 | 613 | 16,443 | 264 | 0.88 | 0.75 | 1.04 |
| *Systolic blood pressure (continuous, per mmHg)* | | | | | | | |
| COVID-19 diagnosis | - | - | - | - | 0.999 | 0.997 | 1.001 |
| COVID-19 hospitalisation^a^ | - | - | - | - | 0.996 | 0.988 | 1.004 |
| COVID-19 death^b^ | - | - | - | - | 0.994 | 0.989 | 0.998 |

Models adjusted for age, sex, ethnicity, deprivation, household size, BMI, smoking status, COVID-19 shielding status, date of suspected COVID-19 diagnosis, diabetes, chronic kidney disease, previous stroke, previous transient ischemic attack, previous myocardial infarction, chronic lung disease, asthma, chronic obstructive pulmonary disease, cancer, antihypertensive and statin prescription.

BP=blood pressure; CI=confidence interval.

^a^Hospital admission within 28 days of positive COVID-19 case or a COVID-19 diagnosis prior to hospital discharge.

^b^Death within 28 days of a COVID-19 diagnosis.

**Table S3.** Subgroup analyses examining the association between blood pressure control and COVID-19 death* according to RAS exposure and time of infection

| **Subgroup** | **BP category** | **Total population** | **Total with outcome** | **Odds ratio** | **95% CI lower** | **95% CI upper** |
| --- | --- | --- | --- | --- | --- | --- |
| RAS medication prescribed | Strict BP control | 8,339 | 266 | 1 (ref) | - | - |
|  | Raised BP | 12,183 | 226 | 0.81 | 0.67 | 0.99 |
|  | Stage 1 uncontrolled BP | 9,448 | 161 | 0.75 | 0.60 | 0.94 |
|  | Stage 2 uncontrolled BP | 2,490 | 51 | 0.94 | 0.67 | 1.32 |
| Other antihypertensives prescribed | Strict BP control | 2,484 | 59 | 1 (ref) | - | - |
|  | Raised BP | 3,461 | 45 | 1.00 | 0.63 | 1.58 |
|  | Stage 1 uncontrolled BP | 2,693 | 31 | 0.74 | 0.45 | 1.21 |
|  | Stage 2 uncontrolled BP | 573 | 12 | 1.76 | 0.87 | 3.59 |
| First suspected infection  (Jan-Mar 2020) | Strict BP control | 488 | 58 | 1 (ref) | - | - |
|  | Raised BP | 675 | 63 | 0.98 | 0.63 | 1.52 |
|  | Stage 1 uncontrolled BP | 510 | 28 | 0.60 | 0.35 | 1.03 |
|  | Stage 2 uncontrolled BP | 114 | 13 | 1.36 | 0.65 | 2.86 |
| First suspected infection  (Apr-June 2020) | Strict BP control | 6,211 | 270 | 1 (ref) | - | - |
|  | Raised BP | 8,512 | 213 | 0.86 | 0.69 | 1.05 |
|  | Stage 1 uncontrolled BP | 6,586 | 170 | 0.83 | 0.67 | 1.04 |
|  | Stage 2 uncontrolled BP | 1,691 | 49 | 0.98 | 0.69 | 1.39 |
| First suspected infection  (Jul-Aug 2020) | Strict BP control | 5,251 | 7 | 1 (ref) | - | - |
|  | Raised BP | 7,838 | 2 | 0.25 | 0.05 | 1.28 |
|  | Stage 1 uncontrolled BP | 6,077 | 2 | 0.29 | 0.06 | 1.49 |
|  | Stage 2 uncontrolled BP | 1,465 | 2 | 1.02 | 0.16 | 6.34 |

Models adjusted for age, sex, ethnicity, deprivation, household size, BMI, smoking status, COVID-19 shielding status, date of suspected infection, diabetes, chronic kidney disease, previous stroke, previous transient ischemic attack, previous myocardial infarction, chronic lung disease, asthma, chronic obstructive pulmonary disease, cancer, antihypertensive and statin prescription.

BP=blood pressure; CI=confidence interval; RAS=Renin-angiotensin system medications (i.e. ACE inhibitors or angiotensin II receptor blockers)

*Death within 28 days of a COVID-19 diagnosis.

**Table S4.** Post-hoc subgroup analyses examining the association between blood pressure control and COVID-19 death* according to number of antihypertensive medications prescribed

| **Subgroup** | **BP category** | **Total population** | **Total with outcome** | **Odds ratio** | **95% CI lower** | **95% CI upper** |
| --- | --- | --- | --- | --- | --- | --- |
| 0-2 Antihypertensives | Strict BP control | 5,314 | 83 | 1 (ref) | - | - |
|  | Raised BP | 8,454 | 62 | 0.80 | 0.55 | 1.18 |
|  | Stage 1 uncontrolled BP | 6,221 | 60 | 0.90 | 0.61 | 1.33 |
|  | Stage 2 uncontrolled BP | 1,376 | 16 | 1.68 | 0.91 | 3.12 |
| 3+ Antihypertensives | Strict BP control | 6,636 | 252 | 1 (ref) | - | - |
|  | Raised BP | 8,571 | 216 | 0.85 | 0.69 | 1.05 |
|  | Stage 1 uncontrolled BP | 6,952 | 140 | 0.71 | 0.56 | 0.89 |
|  | Stage 2 uncontrolled BP | 1,894 | 48 | 0.91 | 0.65 | 1.29 |

Models adjusted for age, sex, ethnicity, deprivation, household size, BMI, smoking status, COVID-19 shielding status, date of suspected infection, diabetes, chronic kidney disease, previous stroke, previous transient ischemic attack, previous myocardial infarction, chronic lung disease, asthma, chronic obstructive pulmonary disease, cancer, antihypertensive and statin prescription.

BP=blood pressure; CI=confidence interval

*Death within 28 days of a COVID-19 diagnosis.
