# Supplementary material for: Association Between Blood Pressure Control and Coronavirus Disease 2019 Outcomes in 45 418 Symptomatic Patients With Hypertension: An Observational Cohort Study
Source: Hypertension. 2020 Dec 16;77(3):846–55. doi: 10.1161/HYPERTENSIONAHA.120.16472 (PMC7884248; doi:10.1161/HYPERTENSIONAHA.120.16472)
Supplement: Supplementary file 2 [file hyp-77-846-s002.docx]

**The association between blood pressure control and Coronavirus Disease 2019 outcomes in 45,418 symptomatic patients with hypertension: An observational cohort study**

Short title: Blood pressure control and COVID-19 outcomes

James P. Sheppard, *PhD*^1^ Brian D. Nicholson, *DPhil* ^1^ Joseph Lee, *MRCGP* ^1^ Dylan McGagh, *BSc*^1^ Julian Sherlock, *BA*^1^ Constantinos Koshiaris, *DPhil*^1^ Jason Oke, *DPhil* ^1^ Nicholas R Jones, *MSc*^1^ William Hinton, *MSc*^1^ Laura Armitage, *MB BCh*^1^ Oliver Van Hecke, MRCGP^1^ Sarah Lay-Flurrie, *DPhil*^1^ Clare R. Bankhead, *DPhil* ^1^ Harshana Liyanage, *PhD*^1^ John Williams, *MSc*^1^ Filipa Ferreira, *PhD*^1^ Michael D. Feher, *MD*^1^ Andrew J. Ashworth, MRCGP^2^ Mark P. Joy, *PhD*^1^ Simon de Lusignan*, *MD*^1^ FD Richard Hobbs, *FMedSci**^1^

^1^Nuffield Department of Primary Care Health Sciences, University of Oxford, Oxford, UK

^2^Bonhard Medical, Bonhard House, Bo’ness, UK

*Joint senior author

**Corresponding authors:** Dr James Sheppard and Prof Richard Hobbs

**Email:** [james.sheppard@phc.ox.ac.uk](mailto:james.sheppard@phc.ox.ac.uk); [richard.hobbs@phc.ox.ac.uk](mailto:richard.hobbs@phc.ox.ac.uk)

**Address:** Nuffield Department of Primary Care Health Sciences, Radcliffe Primary Care Building, Radcliffe Observatory Quarter, University of Oxford, Oxford, OX2 6GG, UK

**Word count:** 5,820 (6,000 word max)

**Number of references:** 28

**Number of tables:** 2

**Number of figures:** 3

**Abstract**

Hypertension has been identified as a risk factor for COVID-19 and associated adverse outcomes. This study examined the association between pre-infection blood pressure (BP) control and COVID-19 outcomes using data from 460 general practices in England. Eligible patients were adults with hypertension who were tested or diagnosed with COVID-19. BP control was defined by the most recent BP reading within 24 months of the index date (1^st^ January 2020). BP was defined as controlled (<130/80mmHg), raised (130/80-139/89mmHg), stage 1 uncontrolled (140/90-159/99mmHg) or stage 2 uncontrolled (≥160/100mmHg). The primary outcome was death within 28 days of COVID-19 diagnosis. Secondary outcomes were COVID-19 diagnosis and COVID-19 related hospital admission. Multivariable logistic regression was used to examine the association between BP control and outcomes. Of the 45,418 patients (mean age 67 years; 44.7% male) included, 11,950 (26.3%) had controlled BP. These patients were older, had more co-morbidities and had been diagnosed with hypertension for longer. A total of 4,277 patients (9.4%) were diagnosed with COVID-19 and 877 died within 28 days. Individuals with stage 1 uncontrolled BP had lower odds of COVID-19 death (OR 0.76, 95%CI 0.62-0.92) compared to patients with well-controlled BP. There was no association between BP control and COVID-19 diagnosis or hospitalisation. These findings suggest BP control may be associated with worse COVID-19 outcomes, possibly due to these patients having more advanced atherosclerosis and target organ damage. Such patients may need to consider adhering to stricter social-distancing, to limit the impact of COVID-19 as future waves of the pandemic occur.

**Word count:** 250 (250 max)

**Key words:** COVID-19, SARS-CoV-2, high blood pressure, electronic health records, mortality, hospital admission

**Introduction**

Coronavirus Disease 2019 (COVID-19) is caused by severe acute respiratory syndrome coronavirus 2 (SARS-CoV-2) and has spread rapidly across the globe resulting in significant restrictions on daily life for millions, serious health complications and death.^1^ Over the past six months, studies have identified common co-morbidities in patients with COVID-19, including hypertension and cardiovascular disease,^2^ which increase the likelihood of serious complications such as hospitalisation and death.^3-5^

One initial proposed explanation for the association between hypertension and COVID-19 was that the SARS-CoV-2 virus enters cells in the lung via angiotensin converting enzyme 2 (ACE2) receptors.^6^ People with hypertension are more likely be prescribed medications (such as angiotensin converting enzyme inhibitors or angiotensin II receptor blockers) which upregulate expression of ACE2 and therefore increase patient susceptibility to SARS-CoV-2 cell entry.^7,8^ However, this theory has since been dismissed^9,10^ with some more recent studies suggesting that the prescription of renin-angiotensin system (RAS) medications may in fact protect against SARS-CoV-2 infection^11^ and COVID-19 related death.^12^

Most recently, a study based on data from hospitals in China suggested that it is higher blood pressure, not specific medication use, which is an important independent risk factor for complications such as heart failure in COVID-19 patients with hypertension.^13^ Establishing whether this is also the case for hypertensive patients living in the community is important, since the focus on routine chronic disease management has reduced during the pandemic.^14^ Based on previous studies,^3-5,13,15-17^ we hypothesised that uncontrolled blood pressure would be associated with worse COVID-19 outcomes for hypertensive patients with suspected COVID-19. We used the electronic health records from primary care to test this hypothesis and examined the association between blood pressure control and SARS-CoV-2 infection, COVID-19 related hospitalisation and death.

**Methods**

*Design*

This study used a retrospective observational cohort study design, utilising electronic health records from general practices in England contributing to the Oxford Royal College of General Practitioners Clinical Informatics Digital Hub (ORCHID).^18-20^ The ORCHID hub is representative of patients attending English primary care across urban and non-urban practices.^19^ The protocol for this study was approved by Royal College of General Practitioners Research Surveillance Centre scientific advisory committee and received ethical approval from the University of Oxford, Medical Sciences Interdivisional Research Ethics Committee (ref: R54893/RE001). Because of the sensitive nature of the data collected for this study, requests to access the dataset from qualified researchers trained in human subject confidentiality protocols may be sent to the RCGP RSC at [MedicalDirectorRSC@rcgp.org.uk](mailto:MedicalDirectorRSC@rcgp.org.uk).

*Study population*

This study examined patients aged 18 years and older, with a coded history of hypertension and registered at general practices in England contributing to ORCHID. Early on in the pandemic, many people are thought to have contracted COVID-19 without realising or being tested.^21^ The present analyses therefore focused on individuals tested for and/or who had a clinical diagnosis of COVID-19 to minimise bias from incomplete outcome ascertainment (i.e. to avoid missing patients who experienced relevant outcomes but were not tested for COVID-19). This information was derived from a newly developed COVID-19 ontology^22^ which uses coded information in an individual’s electronic health record to determine their COVID-19 status. Patients were classified as either not diagnosed with COVID-19 (negative virology test for SARS-CoV-2) or diagnosed with COVID-19 (based on a diagnostic code for COVID-19 or a positive virology test for SARS-CoV-2). All patients entered the cohort on the 1^st^ January 2020 (index date) and were followed until 31^st^ August 2020.

*Exposures*

The primary exposure of interest in this study was blood pressure control at the index date (1^st^ January 2020). This was defined according to the most recently recorded blood pressure in a patient’s electronic health record (within 24 months of the index date). Since readings were taken from routine electronic health records, the exact method of measurement would have varied between patients and was not captured in the record itself. A period of up to 24 months was chosen to maximise the number of participants that could be included in the complete-case analysis. Blood pressure control was specified as a categorical variable according to clinical guidelines,^23^ consisting of controlled (readings <130/80 mmHg), raised (readings between 130/80-139/89 mmHg), stage 1 uncontrolled (readings between 140/90-159/99 mmHg) and stage 2 uncontrolled blood pressure (readings ≥160/100 mmHg). Sensitivity analyses examined blood pressure control defined as a binary variable (readings ±140/90 mmHg), using systolic blood pressure as a continuous variable and using a categorical variable defined by estimating the mean of up to 25 readings taken within the 24 months prior to the index date.

*Outcomes*

The primary outcome in this analysis was death within 28 days of a COVID-19 diagnosis recorded in the patient’s electronic health record, as per the current definition of COVID-19-related death in the UK.^24^ This conservative definition was used because the longer the interval between diagnosis and death, the more likely non-COVID deaths could occur and be misclassified as being COVID-related. Secondary outcomes were COVID-19 diagnosis and hospital admission related to COVID-19. The latter was defined as either a hospital admission within 28 days of COVID-19 diagnosis or a COVID-19 diagnostic code being entered into the medical records after hospital admission but prior to discharge. No linked secondary care or death registry data were available for this analysis, so all outcomes were based on codes entered into the primary care electronic health record.

*Covariates*

All analyses were adjusted for covariates thought to predict COVID-19 outcomes as determined by the previous literature.^1,5,11^ These were age at index date, sex, ethnicity, indices of social deprivation (IMD quintile), number of people within the household, smoking status (current, ex or never smoked), coded as being on the COVID-19 shielding list (due to co-morbidities) and most recent measure of body mass index (BMI), specified as a continuous variable. Those with missing ethnicity or smoking status were classed as unknown ethnicity or non-smokers respectively. Co-morbidities were defined as those present prior to the index date including asthma, cancer, chronic lung disease, chronic obstructive pulmonary disease (COPD), chronic kidney disease, diabetes, previous myocardial infarction, stroke or transient ischemic attack (TIA). Models were adjusted for the presence of a prescribed cardiovascular medication at the index date. This included all blood pressure lowering medications and statins entered as individual drug classes. Each model was also adjusted for the date at which COVID-19 was first suspected using codes from the COVID-19 ontology.^22^

*Statistical analysis*

Descriptive statistics were used to define the characteristics of the study population. Multivariable logistic regression was used to examine the association between blood pressure control and COVID-19 outcomes. This model was chosen since follow-up was short (8 months) and so the likelihood of censoring due to competing risks or loss to follow-up was low. All models were adjusted for the covariates described above, but interaction terms between covariates were not included. Missing data for IMD, BMI, blood pressure and smoking status were low (<5%), so no attempts were made to impute missing values and a complete case analysis was conducted.

Subgroup analyses were undertaken to examine the association between blood pressure control and COVID-19 related death in young vs. older adults (18-69 years vs. 70+ years), those with diabetes, chronic kidney disease, cardiovascular disease and, those prescribed RAS medications vs. those prescribed other blood pressure lowering medications. Because the availability of testing changed significantly during the study period (and therefore the types of patients receiving such tests might also have changed), further analyses were conducted according to the time period in which patients were first suspected of COVID-19 (i.e. Jan-March, April-June and July-Aug 2020). Post-hoc analyses examined the primary outcome in those prescribed 0-2 antihypertensives vs. those prescribed 3+ antihypertensive medications.

All data are presented as means, medians or odds ratios with 95% confidence intervals (CI) or interquartile range. Analyses were conducted using STATA 14.2 (Statacorp, Texas, United States).

**Results**

The ORCHID database included a total of 4,101,459 active patients, from 460 general practices. A total of 45,418 patients had a history of hypertension, had a blood pressure reading in the preceding 24 months (40,645 [89.5%] had a reading within 12 months of the index date) and were tested for or diagnosed with COVID-19. Overall, patients were aged 67.3±16.0 years, 44.7% were male, and 75.6% were of white ethnicity (table 1). The median household size was 2 people (interquartile range 1 to 4) and 15.9% of patients lived in regions with the highest levels of deprivation (5th quintile of IMD).

There were 11,950 (26.3%) patients with controlled blood pressure, 17,025 (37.5%) with moderately raised blood pressure and 16,443 (36.2%) with uncontrolled blood pressure (stage 1 or above). A higher proportion of patients with raised and uncontrolled blood pressure were of black ethnicity but fewer were coded with COVID-19 shielding status (table 1). Patients with controlled blood pressure were older (71 years vs. 65-67 years) and had been diagnosed with hypertension for least 1.4 years longer than those with raised or uncontrolled blood pressure. They also had more co-morbidities including chronic kidney disease (26.3%), COPD (8.6%), diabetes (29.5%), history of myocardial infarction (9.4%) and stroke or TIA (14.4%).

A total of 4,277 (9.4%) were diagnosed with COVID-19 (including 3,025 [6.7%] with a positive virology test for SARS-CoV-2) (table 2). Across the study population, there were 273 (0.6%) COVID-19 related hospitalisations and 877 (1.9%) COVID-19 related deaths.

*Primary outcome*

In multivariable analyses adjusting for all covariates, individuals with stage 1 uncontrolled blood pressure had lower odds of COVID-19 related death (OR 0.76, 95% CI 0.62 to 0.92) compared to patients with well-controlled blood pressure (<130/80 mmHg) (figure 1). Moderately raised blood pressure and stage 2 or above uncontrolled blood pressure were not associated with COVID-19 related death (raised BP, OR 0.84, 95% CI 0.70 to 1.01; stage 2 or above uncontrolled BP, OR 1.05, 95% CI 0.77 to 1.42; figure 1). Increasing age, male sex, Asian or other ethnicity (compared to white), increasing deprivation, living in a multi-person household, being an ex-smoker and having diabetes were all significant predictors of COVID-19 related death (Table S1, online supplement).

*Secondary outcomes*

There was no association between moderately raised or uncontrolled blood pressure and COVID-19 diagnosis (raised BP, OR 0.94, 95% CI 0.86 to 1.03; stage 1 uncontrolled BP, OR 0.95, 95% CI 0.86 to 1.05; stage 2 uncontrolled BP, OR 1.09, 95% CI 0.93 to 1.27) (figure 1). Further analyses focussing on COVID-19 related hospital admission found no association with blood pressure control (raised BP, OR 0.99, 95% CI 0.73 to 1.34; stage 1 uncontrolled BP, OR 0.91, 95% CI 0.66 to 1.27; stage 2 or above uncontrolled BP, OR 0.58, 95% CI 0.30 to 1.10; figure 1).

*Sensitivity and Subgroup analyses*

Patients had a median of four blood pressure readings (interquartile range 3,7) in the 24 months preceding the index date. In analyses based on the average of these readings, moderately raised blood pressure and stage 1 uncontrolled blood pressure were associated with lower odds of COVID-19 related death, compared to patients with well-controlled blood pressure (figure 2). Stage 1 uncontrolled blood pressure was also associated with lower odds of COVID-19 diagnosis.

Sensitivity analyses including blood pressure control as a binary outcome and systolic blood pressure as a continuous variable confirmed the findings of the primary analysis showing a limited association between uncontrolled blood pressure and COVID-19 related death (Table S2, online appendix). The association between stage 1 uncontrolled blood pressure and COVID-19 related death was only present in older patients (70+ years), those without a history of diabetes, chronic kidney disease or cardiovascular disease (figure 3) and those prescribed RAS medications (Table S3). The findings of the primary analysis were not altered by the time period of first suspected SARS-CoV-2 infection (Table S3). Post-hoc analyses showed the association between stage 1 uncontrolled blood pressure and less COVID-19 related death was only present in patients prescribed three or more antihypertensive medications (Table S4).

**Discussion**

*Summary of main findings*

This is the largest study of COVID-19 outcomes in community-dwelling patients with hypertension conducted to date. Across 45,418 patients with hypertension and suspected COVID-19, those with recent stage 1 uncontrolled blood pressure had lower odds of COVID-19 related death compared to patients with well-controlled blood pressure. There was no association between moderately raised blood pressure or stage 2 uncontrolled blood pressure and COVID-19 related death. These findings were robust to sensitivity analyses and contrary to our hypothesis, that raised or uncontrolled blood pressure would be associated with worse COVID-19 outcomes. In analyses defining blood pressure control over a longer period of time (across two years prior to the index date), both moderately raised and stage 1 uncontrolled blood pressure were associated with lower odds of COVID-19 related death.

Patients with strictly controlled blood pressure were older, had more co-morbidities and had been diagnosed with hypertension for longer. A possible explanation for the observed associations is that patients with strict blood pressure control had more advanced atherosclerosis compared to those with moderately raised and uncontrolled blood pressure. This interpretation is supported by our observation of a higher prevalence of target organ damage (including chronic kidney disease, myocardial infarction, stroke and TIA) in those with strictly controlled hypertension and other data suggesting that COVID-19 and cardiovascular disease have a bidirectional relationship.^2^ These findings suggest those with long-term controlled blood pressure may need to consider stricter social distancing to limit the impact of COVID-19 as future waves of the pandemic occur.

*Strengths and limitations*

This is the largest study examining the association between blood pressure and COVID-19 outcomes conducted in community dwelling patients with hypertension. The ORCHID hub^20^ is capable of weekly data downloads permitting some of the most timely and up-to-date analysis of primary care data in the world. Data from secondary care are not available in such a timely manner in the UK and for this analysis, it was not possible to link primary care data to hospital databases or the national death registry. As a result, it is possible that the total number of COVID-19 related outcomes (particularly hospital admissions, which were lower than anticipated) may have been underestimated. The quality of coding of hospitalisation in primary care records is likely to vary between primary care providers^25^ and so we implemented a strict definition of COVID-19 related admissions (within 28 days of diagnosis) which may also have resulted in some relevant outcomes being missed.

Further, because the data used here included patients tested for COVID-19 within a week of conducting the analysis, some recently infected patients may have gone on to have hospital admissions or die but these outcomes would not have been captured in this analysis. We would not expect systematic differences in the recording of COVID-19 outcomes depending on an individual’s blood pressure level, so the impact of these potential missing outcome data is likely to be small.

This was an observational study using data from routine electronic health records. As such, the main exposure (blood pressure) was based on measurements taken in routine clinical practice. One-off measurements taken in this setting may not accurately reflect the underlying blood pressure of each patient, leading to the potential for classification bias. However, sensitivity analyses examining blood pressure based on the mean of up to 25 readings taken across the preceding 24-month period showed similar findings, suggesting this bias may not have had an important influence on the results.

People with very low blood pressure, on multiple antihypertensive medications have been shown to be at increased risk of mortality.^26^ However, whilst some of these may have been captured in the strictly controlled blood pressure group, we do not think there were sufficient numbers to notably alter the findings.

Finally, rates of COVID-19 testing have changed significantly during the pandemic which may have affected ascertainment of COVID-19 cases and the type of patient included in the analysis cohort (potential selection bias). To mitigate for this, we implemented a COVID-19 ontology^22^ which allowed us to identify and include patients with coded COVID-19 diagnosis, but who did not receive a confirmatory virology test. Date of first suspected SARS-CoV-2 infection was adjusted in the analysis and subgroup analyses by time period and did not show a difference in the association between blood pressure control and COVID-19 outcomes, suggesting any changes in the ascertainment of cases or potential selection bias did not have a large impact on the main findings.

*Comparison with previous literature*

Hypertension has previously been shown to be a risk factor for worse COVID-19 outcomes,^6^ and there are some data to suggest that patients admitted to intensive care with COVID-19 have higher blood pressure compared to those who do not have COVID-19.^15,16^ A recent study from China showed that patients with higher blood pressure during admission to hospital have a higher risk of heart failure but not mortality or intensive care unit admission.^13^ In contrast, the present study found an inverse relationship between recent blood pressure control and COVID-19 related death. This relationship was robust to sensitivity analyses examining blood pressure as a continuous variable and defining blood pressure control using the average of readings taken across the preceding two years. The fact that our study focussed on blood pressure control before COVID-19 diagnosis, rather than during hospitalisation for an infection may explain these discrepant results.

Given the limited data on COVID-19 and related risk factors,^3-5,17^ this finding is entirely novel and not easily explained. It may be a chance statistical finding due to multiple hypothesis testing and future studies should look to confirm the relationship observed in these data. It is also possible that other important risk factors were present in the group with controlled blood pressure (such as congestive heart failure), but not adequately adjusted for in the analysis. Indeed, when this study was conceived, very little was known about what conditions and medications modify the risk of COVID-19 related death and so it was not possible to include them in the dataset and adjust for them in the analysis.

Another possible explanation is that blood pressure control, as defined in the present analyses, is a surrogate marker for underlying atherosclerosis, which in turn is associated with increased odds of COVID-19 outcomes.^2^ This would seem to be backed up by the observation that individuals with controlled blood pressure were older, had more co-morbidities (including target organ damage) and had been diagnosed with hypertension for longer. In the UK, blood pressure treatment targets are lower for people with co-morbidities such as diabetes and chronic kidney disease^27^ and physicians may be more likely to treat hypertension aggressively in high-risk patients with established cardiovascular disease. This would lead to individuals at higher risk of COVID-19 related outcomes being more likely to have controlled blood pressure.

*Implications for clinical practice*

Establishing the association between hypertension control and COVID-19 outcomes has important implications for ongoing management, particularly as future ‘waves’ of the pandemic occur. This analysis suggests that recent, poorly controlled blood pressure does not carry an increased risk of COVID-19 related complications, beyond that of the underlying hypertension. This may be reassuring given that chronic disease management has been de-prioritised during the pandemic.^14^ However, high blood pressure remains a strong risk factor for cardiovascular disease including stroke,^28^ the consequences of which can be comparable or worse than those of COVID-19. Thus, whilst stricter blood pressure control in patients with hypertension does not appear to reduce the risk of COVID-19 complications, physicians should continue to ensure adequate blood pressure control to prevent long-term outcomes such as stroke. They may also attempt to identify and monitor individuals with advanced atherosclerosis, perhaps focusing on those who have had well-controlled blood pressure for a longer period of time or who have been diagnosed with hypertension for many years. These patients may need to consider adhering to stricter social distancing, to limit the impact of COVID-19 as future waves of the pandemic occur.

*Perspectives*

This study found little evidence to support the hypothesis that stricter blood pressure control reduces the risk of complications from COVID-19 in patients with hypertension. Future studies should look to confirm the observation that blood pressure control is associated with increased odds of COVID-19 related death. This may be due to underlying atherosclerosis in these patients and physicians should monitor such patients carefully, as they may need to adhere to stricter social distancing to limit the impact of COVID-19 in future waves of the pandemic.

**Acknowledgements**

**Sources of funding**

This piece of work was not specifically funded but used data from the ORCHID hub, which is partially supported by the University of Oxford Medical Sciences Division Urgent COVID Fund and a discretionary award from the Primary Care Research Trust. All COVID-19 research conducted within ORCHID is supported by Public Health England, the National institute for Health Research (NIHR) Oxford and Thames Valley Applied Research Collaboration. JS is supported by the Wellcome Trust/Royal Society via a Sir Henry Dale Fellowship (ref: 211182/Z/18/Z) and the NIHR Oxford Biomedical Research Centre. NJ is supported by a Wellcome Trust Doctoral Research Fellowship (ref: 203921/Z/16/Z). FDRH acknowledges part-funding from the NIHR School for Primary Care Research, the NIHR Collaboration for Leadership in Health Research and Care (CLARHC) Oxford, the NIHR Oxford Biomedical Research Centre (BRC, UHT), and the NIHR Oxford Medtech and In-Vitro Diagnostics Co-operative (MIC). CRB is supported by the NIHR Oxford Biomedical Research Centre and the NIHR Thames Valley Applied Research Collaborative.

**Disclosures**

The authors declare no conflicts of interest.

**References**

1. Guan W-j, Ni Z-y, Hu Y, et al. Clinical Characteristics of Coronavirus Disease 2019 in China. *New England Journal of Medicine.* 2020.

2. Nishiga M, Wang DW, Han Y, Lewis DB, Wu JC. COVID-19 and cardiovascular disease: from basic mechanisms to clinical perspectives. *Nature Reviews Cardiology.* 2020;17(9):543-558.

3. Wu Z, McGoogan JM. Characteristics of and Important Lessons From the Coronavirus Disease 2019 (COVID-19) Outbreak in China: Summary of a Report of 72 314 Cases From the Chinese Center for Disease Control and Prevention. *JAMA.* 2020.

4. Zhou F, Yu T, Du R, et al. Clinical course and risk factors for mortality of adult inpatients with COVID-19 in Wuhan, China: a retrospective cohort study. *The Lancet.* 2020.

5. Williamson EJ, Walker AJ, Bhaskaran K, et al. Factors associated with COVID-19-related death using OpenSAFELY. *Nature.* 2020;584(7821):430-436.

6. Zhou P, Yang XL, Wang XG, et al. A pneumonia outbreak associated with a new coronavirus of probable bat origin. *Nature.* 2020;579(7798):270-273.

7. Patel AB, Verma A. COVID-19 and Angiotensin-Converting Enzyme Inhibitors and Angiotensin Receptor Blockers: What Is the Evidence? *JAMA.* 2020.

8. Sparks MA HS, et al.,. The Coronavirus Conundrum: ACE2 and Hypertension Edition. 2020. Accessed 25/03/2020.

9. Fosbøl EL, Butt JH, Østergaard L, et al. Association of Angiotensin-Converting Enzyme Inhibitor or Angiotensin Receptor Blocker Use With COVID-19 Diagnosis and Mortality. *JAMA.* 2020;324(2):168-177.

10. Mehta N, Kalra A, Nowacki AS, et al. Association of Use of Angiotensin-Converting Enzyme Inhibitors and Angiotensin II Receptor Blockers With Testing Positive for Coronavirus Disease 2019 (COVID-19). *JAMA Cardiology.* 2020.

11. Hippisley-Cox J, Young D, Coupland C, et al. Risk of severe COVID-19 disease with ACE inhibitors and angiotensin receptor blockers: cohort study including 8.3 million people. *Heart.* 2020:heartjnl-2020-317393.

12. Gao C, Cai Y, Zhang K, et al. Association of hypertension and antihypertensive treatment with COVID-19 mortality: a retrospective observational study. *European Heart Journal.* 2020;41(22):2058-2066.

13. Ran J, Song Y, Zhuang Z, et al. Blood pressure control and adverse outcomes of COVID-19 infection in patients with concomitant hypertension in Wuhan, China. *Hypertension Research.* 2020.

14. Wright A, Salazar A, Mirica M, Volk LA, Schiff GD. The Invisible Epidemic: Neglected Chronic Disease Management During COVID-19. *Journal of general internal medicine.* 2020:1-2.

15. Chen T, Wu D, Chen H, et al. Clinical characteristics of 113 deceased patients with coronavirus disease 2019: retrospective study. *BMJ.* 2020;368:m1091.

16. Huang C, Wang Y, Li X, et al. Clinical features of patients infected with 2019 novel coronavirus in Wuhan, China. *Lancet (London, England).* 2020;395(10223):497-506.

17. de Lusignan S, Joy M, Oke J, et al. Disparities in the excess risk of mortality in the first wave of COVID-19: Cross sectional study of the English sentinel network. *Journal of Infection.* 2020:4817.

18. de Lusignan S, Lopez Bernal J, Zambon M, et al. Emergence of a Novel Coronavirus (COVID-19): A Protocol for Extending Surveillance Used by the Royal College of General Practitioners (RCGP) Research and Surveillance Centre (RSC) and Public Health England (PHE). *JMIR Public Health and Surveillance.* 2020.

19. Correa A, Hinton W, McGovern A, et al. Royal College of General Practitioners Research and Surveillance Centre (RCGP RSC) sentinel network: a cohort profile. *BMJ Open.* 2016;6(4):e011092.

20. de Lusignan S, Jones N, Dorward J, et al. The Oxford Royal College of General Practitioners Clinical Informatics Digital Hub: Protocol to Develop Extended COVID-19 Surveillance and Trial Platforms. *JMIR Public Health Surveill.* 2020;6(3):e19773.

21. Li R, Pei S, Chen B, et al. Substantial undocumented infection facilitates the rapid dissemination of novel coronavirus (SARS-CoV-2). *Science.* 2020;368(6490):489-493.

22. de Lusignan S, Liyanage H, McGagh D, et al. In-pandemic development of an application ontology for COVID-19 surveillance in a primary care sentinel network. *JMIR Preprints.* 2020.

23. Williams B, Mancia G, Spiering W, et al. 2018 ESC/ESH Guidelines for the management of arterial hypertension. *Eur Heart J.* 2018;39(33):3021-3104.

24. UK Government. Coronavirus (COVID-19) in the UK: Deaths in the United Kingdom. 2020; <https://coronavirus.data.gov.uk/details/deaths>.

25. Herrett E, Shah AD, Boggon R, et al. Completeness and diagnostic validity of recording acute myocardial infarction events in primary care, hospital care, disease registry, and national mortality records: cohort study. *Bmj.* 2013;346:f2350.

26. Benetos A, Labat C, Rossignol P, et al. Treatment With Multiple Blood Pressure Medications, Achieved Blood Pressure, and Mortality in Older Nursing Home Residents: The PARTAGE Study. *JAMA Intern Med.* 2015;175(6):989-995.

27. National Guideline Centre. National Institute for Health and Care Excellence. In: *Hypertension in adults: diagnosis and management [NICE guideline 136].* London: Royal College of Physicians (UK); 2019.

28. Lewington S, Clarke R, Qizilbash N, Peto R, Collins R. Age-specific relevance of usual blood pressure to vascular mortality: a meta-analysis of individual data for one million adults in 61 prospective studies. *Lancet (London, England).* 2002;360(9349):1903-1913.

**Novelty and Significance**

*What Is New?*

- This is the largest study of COVID-19 related outcomes in community dwelling patients with hypertension.
- Findings show that patients with recent stage 1 uncontrolled blood pressure had lower odds of COVID-19 death compared to patients with well-controlled blood pressure.

*What Is Relevant?*

- Patients with controlled blood pressure were older, had more co-morbidities and had been diagnosed with hypertension for longer.
- These patients may have more advanced underlying atherosclerosis and target organ damage leading to greater risk of COVID-19 complications.

*Summary*

- Hypertensive patients with long-term blood pressure control may need to consider adhering to stricter social distancing to limit the impact of COVID-19 in future waves of the pandemic.

**Figure legends**

**Figure 1.** Primary analysis showing the association between blood pressure control and COVID-19 diagnosis, COVID-19 related hospitalisation and death

Models adjusted for age, sex, ethnicity, deprivation, household size, BMI, smoking status, COVID-19 shielding status, date of suspected COVID-19 diagnosis, diabetes, chronic kidney disease, previous stroke, previous transient ischemic attack, previous myocardial infarction, chronic lung disease, asthma, chronic obstructive pulmonary disease, cancer, antihypertensive and statin prescription.

BP=blood pressure; OR=odds ratio; CI=confidence interval.

^a^Hospital admission within 28 days of positive COVID-19 case or a COVID-19 diagnosis prior to hospital discharge.

^b^Death within 28 days of a COVID-19 diagnosis.

**Figure 2.** Sensitivity analyses examining the association between blood pressure control (defined by the mean of all blood pressure readings in the preceding 2 years) and COVID-19 diagnosis, COVID-19 related hospitalisation and death

Models adjusted for age, sex, ethnicity, deprivation, household size, BMI, smoking status, COVID-19 shielding status, date of suspected COVID-19 diagnosis, diabetes, chronic kidney disease, previous stroke, previous transient ischemic attack, previous myocardial infarction, chronic lung disease, asthma, chronic obstructive pulmonary disease, cancer, antihypertensive and statin prescription.

BP=blood pressure; OR=odds ratio; CI=confidence interval.

^a^Hospital admission within 28 days of positive COVID-19 case or a COVID-19 diagnosis prior to hospital discharge.

^b^Death within 28 days of a COVID-19 diagnosis.

**Figure 3.** Subgroup analyses examining the association between blood pressure control and COVID-19 related death according age and selected co-morbidities

Models adjusted for age, sex, ethnicity, deprivation, household size, BMI, smoking status, COVID-19 shielding status, date of suspected COVID-19 diagnosis, diabetes, chronic kidney disease, previous stroke, previous transient ischemic attack, previous myocardial infarction, chronic lung disease,

BP=blood pressure; OR=odds ratio; CI=confidence interval.

**Tables**

**Table 1.** Characteristics of patients with a history of hypertension at the index date

| Characteristic | Total population | | BP controlled (<130/80 mmHg) | | BP raised  (130/80-139/89 mmHg) | | Stage 1 uncontrolled  (140/90-159/99 mmHg) | | Stage 2 uncontrolled  (>160/100 mmHg) | |
| --- | --- | --- | --- | --- | --- | --- | --- | --- | --- | --- |
|  | **Total/mean** | **%/SD** | **Total/mean** | **%/SD** | **Total/mean** | **%/SD** | **Total/mean** | **%/SD** | **Total/mean** | **%/SD** |
| Total population | 45,418 |  | 11,950 |  | 17,025 |  | 13,173 |  | 3,270 |  |
| Age (mean, SD; years) | 67.3 | 16.0 | 70.7 | 16.6 | 65.6 | 15.3 | 66.8 | 15.6 | 65.4 | 17.1 |
| Sex (% male) | 20,301 | 44.7% | 5,013 | 41.9% | 7,802 | 45.8% | 6,040 | 45.9% | 1,446 | 44.2% |
| Ethnicity |  |  |  |  |  |  |  |  |  |  |
| White | 34,334 | 75.6% | 9,175 | 76.8% | 12,792 | 75.1% | 9,951 | 75.5% | 2,416 | 73.9% |
| Black | 1,363 | 3.0% | 269 | 2.3% | 574 | 3.4% | 409 | 3.1% | 111 | 3.4% |
| Asian | 2,319 | 5.1% | 600 | 5.0% | 1,009 | 5.9% | 567 | 4.3% | 143 | 4.4% |
| Mixed Race | 296 | 0.7% | 62 | 0.5% | 125 | 0.7% | 83 | 0.6% | 26 | 0.8% |
| Other | 246 | 0.5% | 58 | 0.5% | 90 | 0.5% | 77 | 0.6% | 21 | 0.6% |
| Unknown | 6,860 | 15.1% | 1,786 | 14.9% | 2,435 | 14.3% | 2,086 | 15.8% | 553 | 16.9% |
| Deprivation  (most deprived IMD quintile) | 7,214 | 15.9% | 2,010 | 16.8% | 2,684 | 15.8% | 2,012 | 15.3% | 508 | 15.5% |
| Household size (median, IQR) | 2 | (1,4) | 2 | (2,6) | 2 | (1,4) | 2 | (1,4) | 2 | (1,4) |
| More than 5 co-habitants | 7,912 | 17.4% | 3,005 | 25.1% | 2,430 | 14.3% | 1,963 | 14.9% | 514 | 15.7% |
| Smoking status |  |  |  |  |  |  |  |  |  |  |
| Non-smoker | 12,605 | 27.8% | 3,251 | 27.2% | 4,759 | 28.0% | 3,678 | 27.9% | 917 | 28.0% |
| Ex-smoker | 27,896 | 61.4% | 7,490 | 62.7% | 10,464 | 61.5% | 8,046 | 61.1% | 1,896 | 58.0% |
| Current smoker | 4,786 | 10.5% | 1,157 | 9.7% | 1,775 | 10.4% | 1,410 | 10.7% | 444 | 13.6% |
| COVID-19 shielding | 5809 | 12.8% | 1,821 | 15.2% | 2,009 | 11.8% | 1,583 | 12.0% | 396 | 12.1% |
| BMI (mean, SD; kg/m^2^) | 29.3 | 6.6 | 27.9 | 6.4 | 29.6 | 6.4 | 29.8 | 6.7 | 30.2 | 7.4 |
| Systolic (mean, SD; mm Hg) | 133.4 | 15.5 | 117.2 | 9.1 | 130.9 | 6.6 | 143.7 | 7.1 | 165.0 | 14.2 |
| Diastolic (mean, SD ; mm Hg) | 77.9 | 10.7 | 68.7 | 7.3 | 78.2 | 7.3 | 82.3 | 9.4 | 92.6 | 13.0 |
| Medical history |  |  |  |  |  |  |  |  |  |  |
| Asthma | 8,236 | 18.1% | 2,082 | 17.4% | 3,153 | 18.5% | 2,399 | 18.2% | 602 | 18.4% |
| Cancer | 6,307 | 13.9% | 1,898 | 15.9% | 2,207 | 13.0% | 1,778 | 13.5% | 424 | 13.0% |
| Chronic lung disease | 3,444 | 7.6% | 1,095 | 9.2% | 1,161 | 6.8% | 941 | 7.1% | 247 | 7.6% |
| Chronic kidney disease | 9,453 | 20.8% | 3,141 | 26.3% | 3,173 | 18.6% | 2,481 | 18.8% | 658 | 20.1% |
| COPD | 3210 | 7.1% | 1,030 | 8.6% | 1,088 | 6.4% | 863 | 6.6% | 229 | 7.0% |
| Diabetes | 11,465 | 25.2% | 3,521 | 29.5% | 4,333 | 25.5% | 2,891 | 21.9% | 720 | 22.0% |
| Myocardial infarction | 2,856 | 6.3% | 1,126 | 9.4% | 927 | 5.4% | 651 | 4.9% | 152 | 4.6% |
| Stroke or TIA | 5,068 | 11.2% | 1,719 | 14.4% | 1,681 | 9.9% | 1,311 | 10.0% | 357 | 10.9% |
| Time since hypertension diagnosis (years) | 12.3 | 9.4 | 13.4 | 9.7 | 12.0 | 9.1 | 12.0 | 9.4 | 11.7 | 10.0 |
| Prescribed medications |  |  |  |  |  |  |  |  |  |  |
| ACE inhibitors | 29,703 | 65.4% | 7,609 | 63.7% | 11,170 | 65.6% | 8,619 | 65.4% | 2,305 | 70.5% |
| ARBs | 12,041 | 26.5% | 3,071 | 25.7% | 4,422 | 26.0% | 3,599 | 27.3% | 949 | 29.0% |
| Calcium channel blockers | 28,101 | 61.9% | 6,868 | 57.5% | 10,328 | 60.7% | 8,624 | 65.5% | 2,281 | 69.8% |
| Thiazides | 17,397 | 38.3% | 4,563 | 38.2% | 6,235 | 36.6% | 5,203 | 39.5% | 1,396 | 42.7% |
| Beta-blockers | 20,563 | 45.3% | 6,028 | 50.4% | 7,438 | 43.7% | 5,622 | 42.7% | 1,475 | 45.1% |
| Alpha blockers | 5,836 | 12.8% | 1,429 | 12.0% | 1,953 | 11.5% | 1,831 | 13.9% | 623 | 19.1% |
| Other antihypertensives | 13,438 | 29.6% | 4,344 | 36.4% | 4,459 | 26.2% | 3,645 | 27.7% | 990 | 30.3% |
| Statins | 26,320 | 58.0% | 7,650 | 64.0% | 9,731 | 57.2% | 7,300 | 55.4% | 1,639 | 50.1% |

IMD=Indices of multiple deprivation; BMI=body mass index; COPD=chronic obstructive pulmonary disease; TIA=transient ischemic attack; ACE=Angiotensin converting enzyme; ARBs=Angiotensin II receptor blocker; BP=Blood pressure; SD=standard deviation.

**Table 2.** Patients being investigated for COVID and experiencing outcomes during follow-up

| COVID outcome | Total population | | BP controlled (<130/80 mmHg) | | BP raised  (130/80-139/89 mmHg) | | Stage 1 uncontrolled  (140/90-159/99 mmHg) | | Stage 2 uncontrolled  (>160/100 mmHg) | |
| --- | --- | --- | --- | --- | --- | --- | --- | --- | --- | --- |
|  | **Total** | **%** | **Total** | **%** | **Total** | **%** | **Total** | **%** | **Total** | **%** |
| SARS-CoV-2 test negative | 41,141 | 90.6% | 10,645 | 89.1% | 15,526 | 91.2% | 12,012 | 91.2% | 2,958 | 90.5% |
| SARS-CoV-2 test positive | 3,025 | 6.7% | 939 | 7.9% | 1,060 | 6.2% | 806 | 6.1% | 220 | 6.7% |
| COVID-19 diagnosis^b^ | 1,252 | 2.8% | 366 | 3.1% | 439 | 2.6% | 355 | 2.7% | 92 | 2.8% |
| COVID-19 related hospital admission^c^ | 273 | 0.6% | 86 | 0.7% | 103 | 0.6% | 73 | 0.6% | 11 | 0.3% |
| COVID-19 related death^d^ | 877 | 1.9% | 335 | 2.8% | 278 | 1.6% | 200 | 1.5% | 64 | 2.0% |

BP=Blood pressure.

^a^Patients who were either exposed to someone with COVID-19, investigated or suspected by the GP due presenting symptoms^22^

^b^Based on a diagnostic code or confirmatory virology test

^c^Hospital admission within 28 days of positive COVID-19 case or a COVID-19 diagnosis prior to hospital discharge.

^d^Death within 28 days of a COVID-19 diagnosis.
