# Supplementary figures and images for: Association Between Blood Pressure Control and Coronavirus Disease 2019 Outcomes in 45 418 Symptomatic Patients With Hypertension: An Observational Cohort Study
Source: Hypertension. 2020 Dec 16;77(3):846–55. doi: 10.1161/HYPERTENSIONAHA.120.16472 (PMC7884248; doi:10.1161/HYPERTENSIONAHA.120.16472)

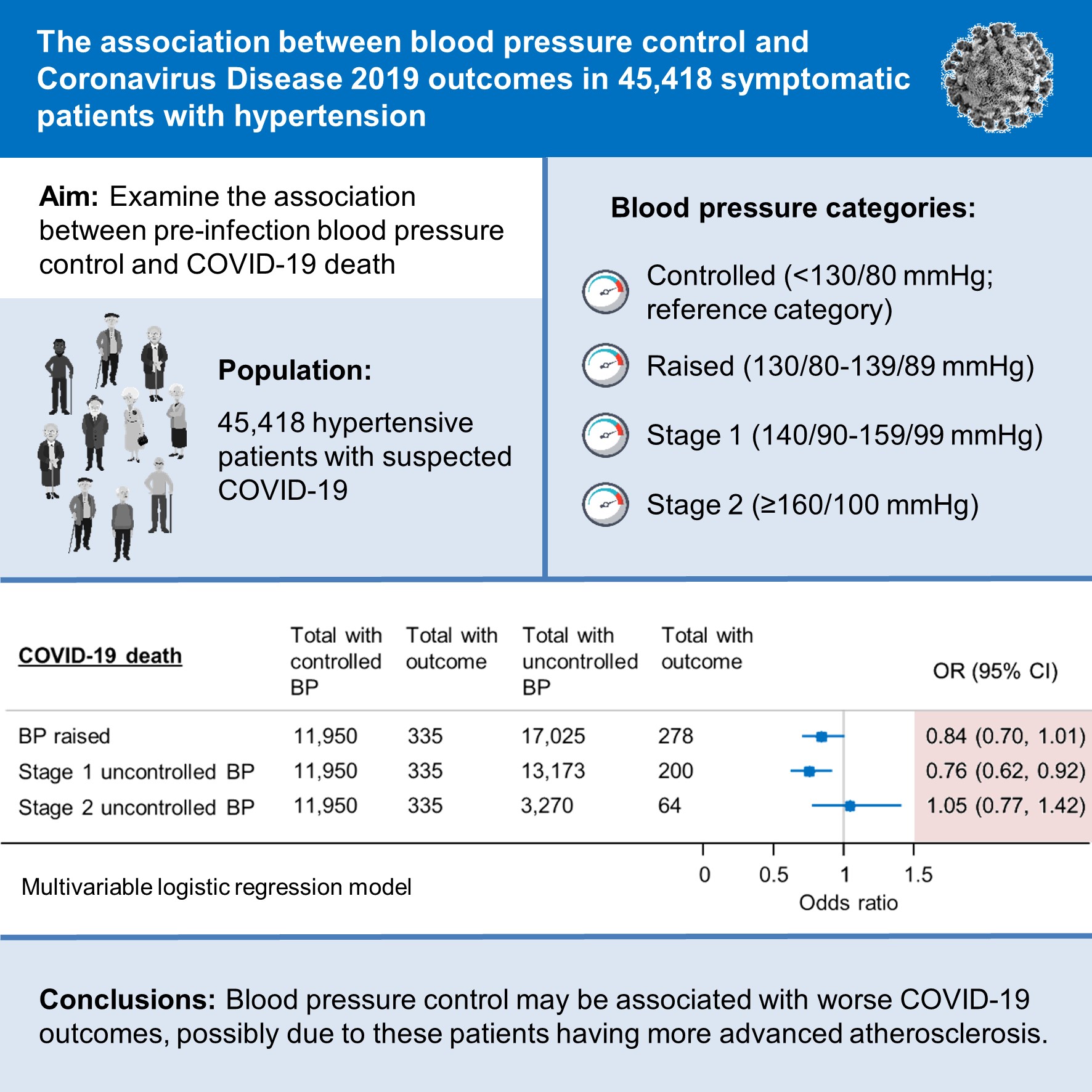

Supplement: Supplementary file 3 [file hyp-77-846-s003.jpg]
